# The Effect of Raw Soybean on Oxidative Status of Digestive Organs in Mice

**DOI:** 10.3390/ijms12128836

**Published:** 2011-12-02

**Authors:** Chunmei Gu, Hongsheng Qu, Lingling Han, Xinxiu Song, Linlin Zhao, Wenfa Lu

**Affiliations:** 1 Institute of Food Science and Engineering, Jilin Agricultural University, Changchun, Jilin 130118, China; E-Mails: jjnong2008@126.com (C.G.); 758635103@qq.com (H.Q.); 511374013@qq.com (L.H.); 519038276@qq.com (X.S.); 630875467@qq.com (L.Z.); 2 Institute of Animal Science and Technology, Jilin Agricultural University, Changchun, Jilin 130118, China

**Keywords:** anti-nutritional factors, digestive organs, free radical, mice, raw soybean

## Abstract

The present study was undertaken to specify the effect of raw soybean on oxidative status of digestive organs in mice. For this purpose, thirty male (C57BL/6J) mice were randomly divided into three groups and fed on different diets as follows: Group 1 was fed on control diet, Group 2 was fed on raw soybean diet and Group 3 was fed on raw soybean diet supplemented with 30 mg/kg cysteamine. After two weeks of feeding, duodenum, liver and pancreas samples were collected to measure oxidative and antioxidative parameters. The results show that ingestion of raw soybean markedly increased contents of superoxide anion and malondialdehyde (MDA) and activity of inducible nitric oxide synthase (iNOS), decreased activity of superoxide dismutase (SOD), T-AOC and content of reduced glutathione (GSH) in digestive organs of mice (*P* < 0.05). In the group fed with raw soybean diet supplemented with cysteamine, oxidative stress was mitigated. However, oxidative parameter levels were still higher than those of control diet-fed group. The present study indicates that ingestion of raw soybean could result in an imbalance between oxidant and antioxidant, and thus induce oxidative stress in digestive organs of mice.

## 1. Introduction

Soy protein is widely used in various forms of human food, such as infant formula, flour, protein concentrate, protein isolate, soy sauce, soy fiber with texture and tofu [1]. However, soy protein contains anti-nutritional factors. It is proved by the production practice that these anti-nutritional factors can lower nutritional value of soybean and even poison livestock, which causes huge economic loss [2–4]. In order to improve utilization efficiency of soybean and its products in human food, soybean anti-nutritional factors have become a focus of domestic and international research in recent years [5–7]. Soybean anti-nutritional factors mainly include trypsin inhibitors, lectin, urease, antigen protein [8], and the harmful role of trypsin inhibitor is one of the most important [1]. Gallaher *et al*. [9] reported when trypsin in the chyme combined with trypsin inhibitor and formed enzyme—inhibitor compound, the activity level of trypsin was reduced and cholecystokinin secretion was increased. Increase of cholecystokinin could stimulate the pancreas to secrete more trypsin into the intestine in order to offset the deficit of trypsin due to trypsin inhibitor. The over-stimulation of pancreatic secretion is bound to cause hypertrophy and hyperplasia of pancreas, also dysfunction or disorder of food digestion and absorption and even dizziness, nausea and diarrhea. However, the conclusions of the current research on these symptoms are still controversial. When the pancreas is stimulated to secrete and synthesize digestive enzymes, a large number of ATP will be required to synthesize purine and pyrimidine and activate amino acids, which are used to further synthesize DNA, mRNA, digestive enzymes and peptide hormones. The generation of ATP is accompanied by free radical production and excessive free radicals can cause lesions to cells through oxidizing DNA, lipids and other components of the cell membrane, and then lead to pathological changes of pancreas and other digestive organs.

On the basis of the above theory, we presume that soybean anti-nutritional factors may increase oxygen free radicals in digestive organs, which might be one of the mechanisms that soybean anti-nutritional factors result in pancreatic hypertrophy and hyperplasia and other harmful effects. The present study is designed to investigate whether raw soybean could increase generation of free radicals and decrease antioxidative ability in digestive organs of mice, at the same time, cysteamine with antioxidant effect was supplemented to raw soybean diet in order to further demonstrate whether raw soybean could induce oxidative stress.

## 2. Materials and Methods

### 2.1. Animals and Diets

The care and use of laboratory animals followed the institutional guidelines of Jilin Agricultural University. Male C57BL/6J mice, weighing 12 ± 2 g at the beginning of the experiment, were given a balanced control diet (200 g casein/kg) for 1 week. After this adaptation period, their weight was 18 ± 5 g. Then all animals were divided randomly into three equal groups of ten animals each and fed on different diets for two weeks as follows: group 1 was fed on the original reference diet; groups 2 was fed on a treatment diet containing raw soybean flour; group 3 was fed on raw soybean diet supplemented with 30 mg/kg cysteamine. Diets were isoenergetic (16.9 MJ/kg) and were given in powdered form. The composition of the diets is shown in Table 1. Mice were housed under a controlled atmosphere (temperature, 23 ± 1 °C; relative humidity, 55 ± 5%; and a fixed 12-h light: dark cycle, light 0700 to 1900 h). They had *ad libitum* access to feed and tap water. Soybean flour was purchased from Jilin Province Agriculture Academy of Science (Jilin, China) and trypsin inhibitor content in the soybean flour measured by the method of Smith *et al.* [10] was 30 g/kg.

### 2.2. Sampling Procedures

At the end of the experimental period, mice were deprived of feed overnight but had free access to deionized water. Mice were sacrificed by decapitation in random order across all three groups and the whole duodenum, liver and pancreas were removed immediately, gently rinsed in ice-cold PBS and then were cut into 50- to 100-mg portions as tissue samples. They were frozen in liquid nitrogen and stored at −80 °C for further treatment. After thawing, tissue samples were homogenized with ice-cold 0.9% NaCl solution and then were centrifuged at 4000 g for 15 min at 4 °C. The supernates were used to determine protein content and antioxidant defense and lipid peroxidation. The protein content was determined using the method of Lowry *et al.* [11].

### 2.3. Analytical Methods

#### 2.3.1. Oxidative Parameters Determination

The level of oxidative stress was determined by specifically measuring superoxide anion, as described by Pick [12]. In this method, electrons were transferred from superoxide anion to nitroblue tetrazolium reduced into formazan, and then reduced nitroblue tetrazolium was monitored spectrophotometrically at 550 nm, that is, OD at 550 nm was obtained to calculate content of superoxide anion expressed in A per milligram protein.

Lipid peroxidation products, thiobarbituric acid reactive substances (TBARS), were measured by a standard method and are expressed as the content of malondialdehyde (MDA) in nanomoles per milligram of protein [13]. In this procedure, 40% trichloroacetic acid and 1.0 mL of 0.2% thiobarbituric acid (TBA) were added to the samples, and then 2% butylated hydroxy-toluene was added to TBA reagent mixture in order to minimize peroxidation during assay procedure. Tubes were then boiled for 15 min and cooled on ice. Seventy percent of trichloroacetic acid was added and tubes were allowed to stand for 20 min, at which time the tubes were centrifuged at 800× *g* for 10 min. The developed color in the supernatant was read at 532 nm on a spectrophotometer.

Inducible nitric oxide synthase(iNOS) activity in digestive organs was determined by Clément *et al.* [14], using commercial kits from Nanjing Jiancheng Bioengineering Institute. In commercial kits, an enzyme linked immunosorbent assay (ELISA) and color reaction of tetramethylbenzidine(TMB) were used, and iNOS activity was related with color change of TMB monitored spectrophotometrically at 450 nm.

#### 2.3.2. Antioxidative Parameters Determination

Total superoxide dismutase (SOD) activity was assayed using hypoxanthine–xanthine oxidase-generated O_2_^−^ to reduce nitrotetrazolium (NBT) monitored spectrophotometrically at 550 nm. Inhibition of NBT reduction to 50% of maximal is defined as 1U of SOD activity and enzyme activity was expressed in units per milligram protein [15].

Reduced glutathione (GSH) was measured by the procedure of Moron *et al*. [16]. In this procedure, reduced GSH reacts with 5, 5 dithiobis-(2-nitrobenzoic acid) to produce a compound that absorbs at 412 nm.

The T-AOC (total anti-oxidative capacity) in digestive organs was determined by Opara *et al*. [17], using commercial kits from Nanjing Jiancheng Bioengineering Institute. In commercial kits, an enzyme linked immunosorbent assay (ELISA) and color reaction of tetramethylbenzidine (TMB) were used, and T-AOC was related with color change of TMB monitored spectrophotometrically at 450 nm.

### 2.4. Statistical Analysis

Data are reported as means ± SD, *n* = 10. Differences between mean values were determined by ANOVA followed by comparisons using the Newman-Keuls multiple range test. Differences with *P* < 0.05 were considered significant.

## 3. Results and Discussion

### 3.1. Oxidative Parameters in Duodenum, Liver and Pancreas of Mice

When the aerobic metabolism of cell progresses normally, O_2_^−^•, the byproduct of mitochondrial respiratory chain, can generate •OH which is the most damaging of the series of free radicals through a series of reactions. These strongly-reactive oxygen free radicals can cause oxidative damage of lipids and proteins (receptors and enzymes) in cell membrane and DNA in the mitochondria, further harming the organism to different extents, and lastly affect the growth, development and aging process.

In the present study, we observed that feeding of the raw soybean diet for 14 days resulted in the development of oxidative stress in experimental mice, as is evident from Table 2. There was a significant (*P* < 0.05) increase in superoxide anion, the precursor of most reactive oxygen species (ROS) and a mediator in oxidative chain reactions, and MDA contents (153% and 482%, 489% and 157%, 300% and 312%) in duodenum, liver and pancreas, respectively, in mice fed raw soybean diet. The increase of MDA concentration is attributed to lipid peroxidation caused by free radicals. These free radicals can cause the lipid oxidation in cell membrane and the increase of lipid protein in plasma [18], therefore, production of lipid oxides is commonly used as evaluation index in toxicology [19,20].

The increase of oxidative parameters contents indicates that the raw soybean diet could lead to the excessive production of reactive oxygen free radicals *in vivo* and lipid peroxidation damage of cell membrane. The possible reason is that trypsin inhibitor, playing a major role in soybean anti-nutritional factors, could combine with trypsin and chymotrypsin to form an enzyme-inhibitor compound when it enters the digestive tract. The formation of this compound results in reduced concentration and activity of trypsin in intestinal tract, which causes negative feedback increase of pancreozymin secretion, and then stimulates pancreatic acinar cells to secrete more enzymes into the intestine in order to offset the inactivation of trypsin inhibitor on digestive enzymes [21]. The secretion and synthesis of enzyme needs ATP provided by mitochondrion, however, by-products of the ATP-generated process are reactive oxygen species O_2_^−^•, H_2_O_2_ and •OH, so production of ATP is accompanied by the large number of free radicals generated, which results in significant increase of free radical level.

In addition, Table 2 also showed that the raw soybean diet-fed group exhibited strikingly high activity of iNOS (inducible nitric oxide synthase) in digestive organs compared to the control diet-fed group (214%, 568% and 445%, respectively). The possible reason is that iNOS can persistently and profusely release nitric oxide (NO). However, NO, on the one hand, makes enzymes, for example SOD and GSH-Px, inactivated by integrating with their thiol-containing (-SH) centre; on the other hand, combines with superoxide anion to generate another free radical, that is, superoxide nitroso free radicals with the strong oxidization which can generate a cytotoxic effect, and then lead to cell necrosis, tissue damage and directly DNA breakage [22]; at the same time causing a large number of antioxidants *in vivo* (for example vitamin E and vitamin C) to be consumed. These harmful roles of NO can lead to an imbalance between production of free radicals and the antioxidant level and aggravate oxidative damage. Therefore, iNOS was used for evaluating the oxidative effect of raw soybean on digestive organs of mice.

In recent years, a supplement of antioxidants in diets has been used in researches of oxidative stress [23,24]. In the present study, this project was also taken. Many studies indicate that cysteamine is an antioxidant [25,26], which may be related to its thiol. However, it has an antioxidant effect only when its dose is lower than 100 mg/kg. So we supplemented the raw soybean diet in the present study with 30 mg/kg cysteamine and we observed that this inclusion significantly reduced the levels of superoxide anion (except in duodenum), MDA and iNOS in digestive organs of mice fed raw soybean diet. This shows that cysteamine plays an antioxidant role in oxidative stress induced by raw soybean, which is attributed to the scavenging ability of its thiol [27] and GSH synthesis stimulated by it [28].

### 3.2. Antioxidant Parameters in Duodenum, Liver and Pancreas of Mice

Table 3 presents the effect of raw soybean on antioxidant parameter levels in digestive organs of mice. Normally, the organism maintains homeostasis of the redox state through regulating a variety of enzymes and non-enzymes system *in vivo* [29–31]. However, this balance will be broken as antioxidative capability is impaired. From the data we could observe that the raw soybean diet-fed group exhibited strikingly lower activity of SOD and GSH content in duodenum, liver and pancreas compared to the control diet-fed group (38% and 62%, 40% and 66%, 55% and 31%, respectively) (Table 3), and cysteamine increased antioxidant levels in tissues of raw soybean diet-fed mice, but mean values were lower than those of G1.

SOD is an important antioxidant enzyme in organisms, and also a natural scavenger of superoxide anion. It can eliminate free radicals produced by cell metabolism and plays an important role in the maintenance of cell membrane structure integrity [32]. In addition, it prevents or lessens damage of reactive oxygen species on cell membrane by scavenging O_2_^−^• and indirectly maintains oxidation and antioxidant balance of cell. GSH, a non-enzymatic antioxidant, is the main component of intracellular antioxidant system and also an endogenous and exogenous antioxidant. It can effectively scavenge free radicals directly or indirectly through enzymatic reactions, for example lipid peroxidation and H_2_O_2_ [33], and promote reproduction of other antioxidants, for instance vitamin E and vitamin C [34,35]. The lack of GSH can result in damage of mitochondrion and increase of ROS [36,37], and meanwhile enhance toxicity effect caused by ROS [38–40]. So in the present study, SOD and GSH were used as enzyme and non-enzymatic antioxidant for evaluating the effect of raw soybean on antioxidant defense system *in vivo*.

In addition, we also observed that T-AOC in duodenum, liver and pancreas was also significantly decreased by 49%, 77% and 70% in the raw soybean diet-fed group compared to the control diet-fed group. Treatment with cysteamine lessened the effect slightly but significantly (*P* < 0.05) in duodenum and pancreas, but not in liver. T-AOC level directly reflects activity of enzymes and level of non-enzymatic antioxidant *in vivo* [41], so it is an important parameter reflecting the antioxidant status of organism [42]. From the data we found that total antioxidative capability of digestive organs in the raw soybean diet-fed group was impaired.

Combined with the oxidative parameters, the above results indicate that raw soybean can weaken enzymatic and non-enzymatic antioxidant defense abilities in digestive organs. The possible reason is that raw soybean induces excessive free radicals production, which destroys the balance between oxidants and antioxidants in digestive organs, consuming a large number of antioxidants.

In a comparison of oxidative and antioxidant parameters results among different tissues for the same treatment, we observed that change regularity of every parameter was different, but the effect of raw soybean on pancreas was comparatively prominent, which was possibly attributed to the trypsin inhibitor in raw soybean. In addition, the possible reason that raw soybean resulted in an increase of free radical levels in duodenum and liver is, on the one hand directly related to other antinutritional factors in raw soybean and on the other hand, indirectly related to the effect of trypsin inhibitor on pancreas.

## 4. Conclusions

In summary, these findings suggest that oxidative stress may occur in digestive organs after mice ingest a raw soybean diet, which was attributed to antinutritional factors in raw soybean that increased free radicals levels and then caused an imbalance between the production of ROS and the capacity of the antioxidant defense system in digestive organs. In addition, supplement with cysteamine in raw soybean diet significantly reduced the oxidative stress in digestive tissues of mice, which further demonstrated that antinutritional factors may induce production of free radicals.

## Figures and Tables

**Table 1:** Composition of the diets (g/kg) a.

	Ingredients	G1	G2	G3
Protein	Casein b	200	-	-
	Soybean protein c	-	200	200
Carbohydrates	Maize starch d	560	435	435
	Soybean carbohydrates c	-	125	125
Fat	Soybean oil d	120	15	15
	Soybean fat c	-	105	105
Fibers	Cellulose	60	42.5	42.5
	Soybean fiber c	-	17.5	17.5
Others	Mineral mix e	40	40	40
	Vitamin mix f	20	20	20
Cysteamine b				0.03

a Diets were semipurified, isoenergetic (16.9 MJ/kg) and were given in powdered form. And the amount of soybean flour in the diet was 50%;

b Shanghai, China;

c Ingredients originating from the soybean flour containing about 210 g fat, 400 g protein and 250 g carbohydrate/kg;

d Changchun, China;

e The mineral mixture provides the following amounts (mg/kg diet): Ca, 4000; K, 2400; Na, 1600; Mg, 400; Fe, 120; trace elements: Mn, 320; Cu, 5; Zn, 18; Co, 0.04; I, 0.02;

f The vitamin mixture provides the following amounts (mg/kg diet): retinol, 12; cholecalciferol, 0.125; thiamin, 40; riboflavin, 30; pantothenic acid, 140; pyridoxine, 20; inositol, 300; cyanocobalamine, 0.1; ascorbic acid, 1600; (dL) α-tocopherol, 340; menadione, 80; nicotinic acid, 200; paraaminobenzoic acid, 100; folic acid, 10; biotin, 0.6; choline, 2720; G1, Group 1, a control diet containing casein; G2, Group 2, a treatment diet containing raw soybean flour; G3, Group 3, raw soybean diet plus 30 mg/kg cysteamine.

**Table 2:** Superoxide anion (A/mg prot) content, MDA content (nmol/mg prot), iNOS activity in digestive organs (U/mg prot).

G	D	L	P

	Superoxide Anion		
G1	0.15 ± 0.01 ^a^	0.18 ± 0.01 ^a^	0.19 ± 0.01 ^a^
G2	0.38 ± 0.01 ^b^	1.06 ± 0.02 ^c^	0.76 ± 0.08 ^c^
G3	0.37 ± 0.01 ^b^	0.91 ± 0.01 ^b^	0.55 ± 0.02 ^b^

	**MDA**		

G1	0.90 ± 0.08 ^a^	3.21 ± 0.19 ^a^	1.39 ± 0.13 ^a^
G2	5.24 ± 0.07 ^c^	8.25 ± 0.59 ^c^	5.73 ± 0.21 ^c^
G3	3.94 ± 0.17 ^b^	6.93 ± 0.18 ^b^	4.53 ± 0.20 ^b^

	**iNOS**		

G1	0.84 ± 0.01 ^a^	0.47 ± 0.04 ^a^	0.31 ± 0.01 ^a^
G2	2.64 ± 0.05 ^c^	3.14 ± 0.16 ^c^	1.69 ± 0.01 ^c^
G3	2.40 ± 0.02 ^b^	2.58 ± 0.12 ^b^	1.54 ± 0.01 ^b^

Values are means ± SD, *n* = 10. Within a column, values without a common superscript significantly differ, *P* < 0.05. MDA, malondialdehyde; iNOS, inducible nitric oxide synthase; G1, Group 1, a control diet containing casein; G1, Group 2, a treatment diet containing raw soybean; G3, Group 3, raw soybean diet plus 0.03 g/kg cysteamine; D, duodenum; L, liver; P, pancreas.

**Table 3:** SOD activity (U/mg prot), GSH content (mg/g prot) and T-AOC (U/mg prot) in digestive organs.

G	D	L	P

	SOD		
G1	103.33 ± 2.93 ^b^	269.14 ± 5.73 ^b^	130.75 ± 2.84 ^c^
G2	63.70 ± 3.59 ^a^	162.04 ± 3.41 ^a^	57.88 ± 2.20 ^a^
G3	66.42 ± 5.75 ^a^	167.43 ± 2.41 ^a^	68.52 ± 3.19 ^b^

	**GSH**		

G1	114.12 ± 1.86 ^b^	202.14 ± 2.79 ^c^	172.36 ± 0.67 ^c^
G2	42.25 ± 2.55 ^a^	66.81 ± 2.91 ^a^	118.29 ± 0.79 ^a^
G3	45.67 ± 0.96 ^a^	84.93 ± 7.33 ^b^	128.27 ± 1.46 ^b^

	**T-AOC**		

G1	11.75 ± 0.03 ^c^	5.62 ± 0.12 ^b^	2.96 ± 0.05 ^c^
G2	5.95 ± 0.09 ^a^	1.24 ± 0.06 ^a^	0.88 ± 0.03 ^a^
G3	6.37 ± 0.04 ^b^	1.44 ± 0.09 ^a^	1.18 ± 0.04 ^b^

Values are means ± SD, *n* = 10. Within a column, values without a common superscript significantly differ, *P* < 0.05. SOD, superoxide dismutase; GSH, reduced glutathione; T-AOC, total antioxidant capacity; G1, Group 1, a control diet containing casein; G2, Group 2, a treatment diet containing raw soybean; G3, Group 3, raw soybean diet plus 0.03 g/kg cysteamine; D, duodenum; L, liver; P, pancreas.
